# Examining the impact of sharing COVID-19 misinformation online on mental health

**DOI:** 10.1038/s41598-022-11488-y

**Published:** 2022-05-16

**Authors:** Gaurav Verma, Ankur Bhardwaj, Talayeh Aledavood, Munmun De Choudhury, Srijan Kumar

**Affiliations:** 1 School of Computational Science and Engineering, College of Computing, Georgia Institute of Technology, Atlanta, GA 30308 USA; 2 Department of Computer Science, Aalto University, 02150 Espoo, Finland; 3 School of Interactive Computing, College of Computing, Georgia Institute of Technology, Atlanta, GA 30308 USA

**Keywords:** Computer science, Risk factors

## Abstract

Misinformation about the COVID-19 pandemic proliferated widely on social media platforms during the course of the health crisis. Experts have speculated that consuming misinformation online can potentially worsen the mental health of individuals, by causing heightened anxiety, stress, and even suicidal ideation. The present study aims to quantify the causal relationship between sharing misinformation, a strong indicator of consuming misinformation, and experiencing exacerbated anxiety. We conduct a large-scale observational study spanning over 80 million Twitter posts made by 76,985 Twitter users during an 18.5 month period. The results from this study demonstrate that users who shared COVID-19 misinformation experienced approximately two times additional increase in anxiety when compared to similar users who did not share misinformation. Socio-demographic analysis reveals that women, racial minorities, and individuals with lower levels of education in the United States experienced a disproportionately higher increase in anxiety when compared to the other users. These findings shed light on the mental health costs of consuming online misinformation. The work bears practical implications for social media platforms in curbing the adverse psychological impacts of misinformation, while also upholding the ethos of an online public sphere.

## Introduction

Misinformation is a threat to the well-being of our society^1,2^. Misinformation is defined as information that has the features of being false or incorrectly presented, whether intentionally or not, determined based on expert evidence and shared with no intention of harm^3^. During the Coronavirus Disease (COVID-19) pandemic, the proliferation of misinformation has had serious and even lethal health ramifications^4^. For example, there were life-threatening consequences resulting from the consumption of Chlorine Dioxide products which were advertised as a COVID-19 cure early on in the pandemic^5^, and unsubstantiated public claims in the U.S. about injecting disinfectant to combat the coronavirus led to a spike in reported accidental poisonings^6^. Recently, misinformation presenting the COVID-19 vaccines as being a cover to implant trackable microchips have fueled vaccine hesitancy^7,8^.

It is no surprise that since long, misinformation is known to be an adversary that accompanies crisis events^9–11^. However, in today’s digital age, with the unparalleled pervasiveness of the Internet and social media, misinformation has an exacerbated impact on people alongside the direct impact of the crisis^10,12^. At the outset of the COVID-19 pandemic, scholars speculated that misinformation can cause new psychiatric symptoms like fear and anxiety in people without mental illness, aggravate the condition of those with pre-existing mental illness, trigger panic attacks, phobias, and obsessive-compulsive disorders, and cause distress to the caregivers of affected individuals^13,14^. For some individuals, COVID-19 related misinformation has been noted to even prevent them from continuing with their normal lifestyle; others have been found to develop manias, where it became so severe, that the person had to be involuntarily committed due to being a danger to themselves or others^15–18^. Broadly speaking, the “infodemic” of false information being shared online that includes potentially harmful advice on “cures” for COVID-19, alarmist reports of anti-Asian propaganda and conspiracy theories together, have been reported to contribute to anxiety and stress for those already affected by the pandemic^19^. Prior studies have shown that misinformation is designed to provoke emotional responses in its consumers like anger, anxiety, and even depression by distorting our thinking, and these emotional responses in turn, further fuel its spread^20^. In addition, survey studies have shown that fear-arousing articles have an affect on people’s emotions, influencing the perceived risk at personal as well as societal levels^21^.

While a majority of existing studies have focused on understanding the role of social media in mediating, amplifying, or otherwise altering the spread of misinformation^22–27^, the aforementioned causal impact of consuming misinformation online on mental health and psychological well-being, such as stress and anxiety, is under-studied. Existing investigations include understanding the cognitive factors that motivate people to share misinformation^28,29^. It has also been demonstrated that prior-anxiety causes increased vulnerability towards misinformation^30–32^. As digital misinformation is becoming pervasive in online social media and accompanies almost every major real-world event^2,33^, it becomes crucial to study how misinformation affects the consequent anxiety levels of those exposed.

To this end, we study the relationship between consumption of misinformation and its impact on mental health through an extensive quantitative analysis on a massive online social media dataset. Specifically, we hypothesize the following causal mechanism: *consumption of misinformation on social media, to the extent indicated by sharing of misinformation, leads to worsened anxiety as expressed online*. We focus our study on sharing of misinformation as it has previously been shown to be a strong, albeit conservative, indicator of high engagement with and consumption of misinformation^34–36^.

A correlation-based analysis can be used to establish an association between sharing of misinformation and experience of anxiety. However, since a variety of factors can precipitate anxiety in individuals, understanding the impact of sharing misinformation on anxiety necessitates establishing a causal relationship between the two phenomena. A causal analysis would involve isolating the effect of sharing misinformation on anxiety levels among such individuals. Experimental approaches have traditionally provided researchers control over assessing the impact of specific factors on outcomes of interest by conducting a randomized controlled trial (RCT), which is the standard for studying causal relationships^37,38^. However, an RCT could be unethical in our context as it would involve exposing individuals to potentially anxiety-inducing misinformation about COVID-19^39^. Randomized controlled trials are also known to be time-consuming and can pose practical limitations and ethical challenges around the scale and implications of the study^37,40,41^. To overcome these challenges, this paper presents a large-scale observational study of information sharing on social media to establish the causal relationship between sharing misinformation and experiencing exacerbated anxiety.

Towards the above goal, we curated a dataset comprising over 80 million Twitter posts made by 76,985 Twitter users from January 1, 2019 to July 15, 2020 (a total of 18.5 months, with the latter 6.5 months spanning into the ongoing pandemic). After appropriate filtering, we analyzed about 30 million Twitter posts from the timelines of 32,290 Twitter users and assigned the users to treatment and control groups based on their tendency to share misinformation—either considerably present or completely absent, respectively. We then compared the resultant anxiety levels of similar users across the two groups, while inferring anxiety levels and misinformation using machine learning techniques.

Our causal analysis indicates a strong positive effect of sharing misinformation on anxiety with medium-large effect size and statistical significance. On computing the extent of this causal effect we find that, ceteris paribus, users who shared COVID-19 related misinformation experienced about two times additional increase in anxiety levels when compared to the increase experienced by users who did not share misinformation. We also find that among users located in the United States, women, racial minorities, and individuals with relatively lower education experienced a higher increase in anxiety compared to men, whites, and individuals with higher education, respectively.

The negative impact of misinformation on our society and crisis events on individuals has attracted the attention of cross-disciplinary researchers from the fields of social science, psychology, and crisis informatics^42–44^. The results from our study not only establish a causal relationship between sharing misinformation and experiencing severe anxiety but also estimate the extent of this causal impact. The insights from our study can contribute to preventive and corrective approaches to mitigate the adverse effects of misinformation.

## Methods

**Figure 1:**
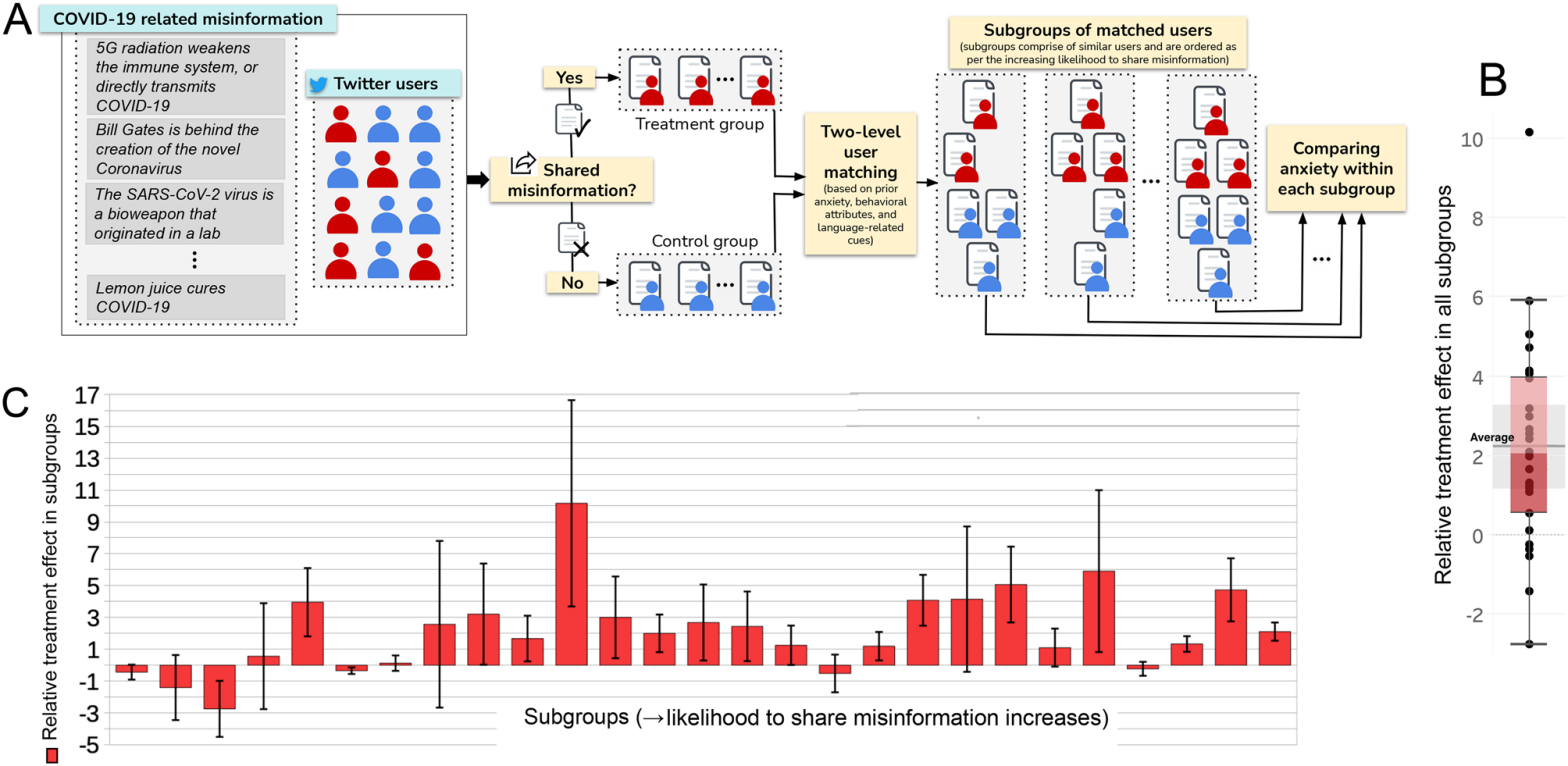
Causal inference methodology (**A**) and the effect of sharing misinformation on experiencing anxiety—overall distribution (**B**) and subgroup-wise values (**C**). We illustrate our methodology to study the causal effect of sharing misinformation (treatment) on experiencing heightened anxiety (outcome) (**A**). We identify users who shared considerable COVID-19 misinformation on Twitter and assign them to the treatment group, while assigning the ones who did not share any misinformation to the control group. We then employ a two-level matching strategy to identify similar users across the two groups, using several factors like prior anxiety, other prior mental health indicators, platform-specific behavioral attributes, and language-related cues. Within each subgroup of matched users, we compare the aggregate anxiety levels of treatment and control users using their post-treatment Twitter posts to estimate the effect of sharing misinformation. In **B**, we show a box and whisker plot of relative treatment effect across all subgroups. The average, first and third quartiles, and the $95\%$ confidence interval all lie above 0. The relative treatment effect in each subgroup and the 95% confidence interval are shown in **C**. Values that are $>0$ indicate a positive effect of sharing misinformation on anxiety within that subgroup. The subgroups are ordered as per the increasing likelihood of sharing misinformation (propensity scores). Regardless of the likelihood to share misinformation, in most subgroups, users who shared misinformation experienced exacerbated anxiety when compared to similar users who did not share misinformation.

For this study, it is required to identify the users who shared COVID-19 related misinformation as well as their prior and post anxiety levels. User timelines on Twitter are a valuable source of data to conduct this study as they unobtrusively capture not only when a user shared misinformative post(s) related to COVID-19, but also the posts they made before and after sharing misinformation. These posts can also be used to infer several other behavioral attributes of users, along with prior and post anxiety levels. Figure 1A gives an overview of the adopted methodology for this study.

### Data

We collected COVID-19 related posts from Twitter between January 1, 2019, and July 15, 2020 (for reference, China reported a cluster of COVID-19 cases in Wuhan on December 31, 2019. The first confirmed case of COVID-19 in the United States was reported on January 20, 2020^45^). We started with the dataset curated by Micallef et al.^46^ comprising 76,985 users who made Twitter posts at least once about COVID-19 during the above time period. We adopted filtering steps to remove inactive accounts, which resulted in the timelines of 43,832 Twitter users. The total number of posts in the resulting dataset was around 40 million—with around 21 million posts in the pre-COVID-19 period (i.e., January 1, 2019, to December 30, 2019), and 19 million posts in the post-COVID-19 period (i.e., December 31, 2019, to July 15, 2020). A detailed description of data filtering steps and descriptive statistics are given in Supplementary Information, Sect. 1. Additionally, we analyzed the accounts to identify bots using two different automated methods and found the fraction of bot accounts to be consistently $< 2.0\%$ in all subcategories, indicating that our study focuses on the behavior of humans; more details regarding the bot analysis are presented in Supplementary Information, Sect. 5.

The two variables of interest in this paper are misinformation and anxiety. Since we do not have ground-truth data indicating the misinformative-ness of a Twitter post or their anxiety level, we trained two machine learning models to infer these variables.

Misinformation Classifier: We trained a large language model called ULMFiT^47^ using a two-stage fine-tuning approach to classify Twitter posts as misinformative or not. The classifier was trained using the manually-labeled data curated by Micallef et al.^46^ and achieved a precision of 0.90 at a classification threshold of 0.70. Once trained, our classifier detected the following COVID-19 misinformation: (i) the claims about certain vitamins and minerals being effective in preventing or treating the disease, (ii) the claims about gargling with warm water, (iii) the claims attributing the spread of the virus to the 5G technology, (iv) the claims about tap water being a spreading agent, (v) the claims about the virus being a biological weapon, and (vi) the claim that Bill Gates played a role in creating the virus. See Supplementary Information, Sect. 2.1 for details.

Anxiety Scorer: The emotion and language used in Twitter posts can be used to infer feelings of stress and anxiety^48–51^. To this end, we used the classifier developed by Saha et al.^51^ to score the anxiety level of Twitter posts on a scale of 0 to 1 using the predicted class probabilities. The classifier presents an accuracy of about 0.90 on held-out test data from Twitter. Additional details about the design of anxiety scorer are provided in Supplementary Information, Sect. 2.2.

### Creating control and treatment groups

Our causal inference framework requires categorizing Twitter users into two groups—those who shared misinformation (treatment group) and those who did not (control group). We used the misinformation classifier to classify each post in all the user timelines as either misinformative or not. We assigned all the users who shared at least five COVID-19 misinformative posts to the treatment group (this threshold was chosen empirically), and those who did not share any misinformation to the control group. Using this assignment technique, out of the 43,832 users, 1,288 users were assigned to the treatment group and 31,002 users were assigned to the control group. In Supplementary Information, Sect. 4, we describe how varying the minimum number of shared misinformative posts for a user to be assigned to the treatment group affects our final results.

Since our study involves measuring the change in anxiety after misinformation has been shared by a Twitter user, we consider the date when a user shared their first COVID-19 related misinformation as their treatment date. For control group users, we assigned them a placebo date by matching the non-parametric distribution of treatment dates, thereby mitigating the effect of temporal confounds. Additional details are provided in Supplementary Information, Sects. 2.3 and 2.4.

### Estimating the causal effect

We worked within a causal inference framework based on matching, which simulates an RCT setting by controlling for as many covariates as possible^52^. Our approach is built on the potential outcomes framework^52,53^, where we examine whether an outcome is caused by a treatment. For our purposes, the outcome is the anxiety levels reflected in the Twitter posts of users and the treatment is sharing misinformation on Twitter. We employed stratified propensity score analysis^54^ to match the users in the treatment group with the users in the control group based on several behavioral attributes, such as prior-anxiety, Twitter interactions, and linguistic cues (see Supplementary Information, Sect. 2.6 for the description of the matching strategy and Supplementary Information, Sect. 2.7 for assessing the quality of matching). Matching provided subgroups of users who had a similar likelihood to share misinformation and could enable meaningful comparison of resultant anxiety across users within the same subgroup as they possess statistically similar attributes. For a subgroup of matched users, we quantified the effect of sharing misinformation within that subgroup as the difference of increase in post-treatment/placebo anxiety levels ($A_{{{after}}}$) with respect to the pre-treatment/placebo anxiety levels ($A_{{{before}}}$) between users who shared misinformation (*trt*) and those who did not (*ctrl*). We also computed the relative additional increase in the anxiety of users who shared misinformation with respect to the increase in the anxiety of users who did not share misinformation—i.e., in comparison to the increase experienced by control group users, how much additional increase do the misinformation sharers experience in their anxiety; see Eq. (1). Additional details are provided in Supplementary Information, Sect. 2.8.

1 $$\begin{aligned} TE_{i}^{{{rel}}} = \frac{({{A}_{{{{after}}}}^{{{trt}}} - {A}_{{{before}}}^{{{trt}}}}) - ({{A}_{{{after}}}^{{{ctrl}}}} - {A}_{{{before}}}^{{{ctrl}}})}{{{{A}_{{{after}}}^{{{ctrl}}}} - {A}_{{{before}}}^{{{ctrl}}}}}. \end{aligned}$$

## Results

Figure 1B and C presents the estimate of the effect of sharing misinformation on an individual’s anxiety. We find the overall relative treatment effect to be 2.011, which demonstrates that in comparison to the increase in anxiety experienced by individuals in the control group, misinformation sharers experienced about two times additional increase in their anxiety (Fig. 1B). Additionally, regardless of users’ likelihood to share misinformation, the users who shared misinformation experienced exacerbated anxiety when compared to those who did not (Fig. 1C). We find that the average Cohen’s *d* between the distributions of post-treatment and post-placebo anxiety levels across all groups of matched users is 0.59, indicating a medium to large effect size. An unequal variances (Welch’s) t-test on distributions of post-treatment and post-placebo anxiety outcomes further revealed that the effect is statistically significant ($t< [-0.31, 7.47]; P < .01$).

As we discuss in Supplementary Information, Sect. 4, the results presented in this work are not sensitive to variations in experimental design choices, such as the minimum count of misinformative posts shared by a user for the assignment to the treatment group and the Jaccard index for linguistic matching. Notably, we find that as we increase the minimum number of misinformative posts shared by a user for them to be assigned to the treatment group, the observed treatment effect on anxiety increases. Specifically, we find the values of $TE^{{{rel}}}$ to be 1.082 ($P < .01$) and 4.639 ($P < .01$) for thresholds of 3 and 7 shared misinformative posts, respectively. We also discuss the negligible contribution of bot accounts on Twitter towards the results of this study (Supplementary Information, Sect. 5) and the validity of Stable Unit Treatment Values Assumption (SUTVA) in the context of our causal inference framework (Supplementary Information, Sect. 6).

### Socio-demographic analysis

To understand how the causal effect of sharing misinformation on anxiety varies across various socio-demographic dimensions, such as sex, race, and education level, we conducted a series of follow-up analyses on US-based treatment-group users (*N*=762) and US-based control-group users (*N*=1198). Since Twitter does not provide any affordances to allow individuals to self-report their sex, race, and education level, we inferred these socio-demographic attributes following techniques used in prior work in social computing^55–57^. See Supplementary Information, Sect. 3.1 for further details about these inference methods.

### Sex and race

**Figure 2:**
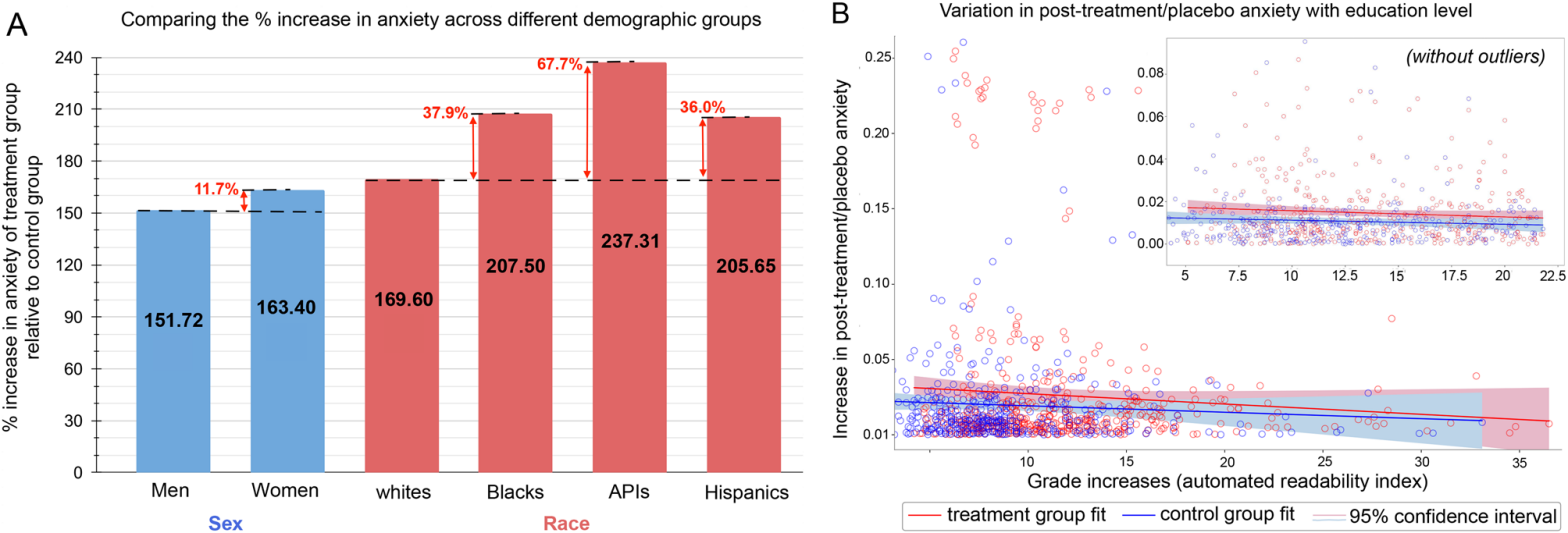
Results from socio-demographic analysis; relative increase in anxiety levels with respect to sex and race (**A**), and education level (**B**). We show the variation in experienced anxiety across different demographic axes in **A**. Each bar represents the percentage increase in post-treatment anxiety levels of individuals in the treatment groups with respect to their control group counterparts. We observe that women and racial minorities are more vulnerable to experiencing exacerbated anxiety as a result of sharing misinformation when compared to men and whites, respectively. The variation in experienced anxiety as a function of automated readability index is shown in **B** (higher ARI corresponds to higher education level). Each circle represents a user in our analysis; the lines of best fit were obtained using ordinary least squares regression. Shaded regions represent the $95\%$ confidence intervals for the treatment and control groups. The trends suggest that higher education level acts as a cushion against the effect of sharing misinformation on experiencing exacerbated anxiety. In inset, we observe similar trends after removing the outliers (i.e., outside of the $\mu \pm 2\sigma$ range).

To infer the sex and race of Twitter users who are located in the U.S., we compared the first and last names of individuals against the U.S. Social Security Administration database and the 2010 U.S. census data, respectively. We compared the increase in anxiety experienced by users of a certain demographic category in the treatment group against the increase in anxiety of control group users who belong to the same demographic category—i.e., women in the treatment group versus women in the control group, and so on. As Fig. 2A illustrates, we find that women in the treatment group experienced 163.4% increase in the anxiety when compared to the control group ($P<.05$), while men experienced an increase of 151.72% ($P<.05$). Furthermore, we also find that in comparison to the 169.6% increase in anxiety experienced by whites in the treatment group ($P<0.01$) with respect to their control group counterparts, Blacks experienced 207.5% increase ($P<.01$), Asian/Pacific Islanders (APIs) experienced 237.31% increase ($P<.05$), and Hispanics experienced 205.65% increase ($P<.1$). These results show that women and racial minorities in the U.S. are more vulnerable to experiencing exacerbated anxiety after sharing misinformation than men and whites, respectively.

### Education level

We inferred the education level of Twitter users in the U.S. by quantifying the readability of their Twitter posts using automated readability index (ARI). ARI is known to produce an approximate representation of the U.S. grade level needed to comprehend the text^58^ and has been used in several prior works to infer the education level of Twitter users^59–61^. Figure 2B shows the variation of the increase in anxiety experienced by the treatment versus control group users in the U.S. with the ARI computed on their Twitter posts. We find that the slope of the best-fit lines (found using ordinary least squares regression; see Supplementary Information, Sect. 3.1 for details) for both the treatment and control group users is negative, indicating that users with higher education levels experienced lesser anxiety when compared to users with lower education. Interestingly, since the slope for the treatment group users is steeper than that for control group users ($- 7.1 \times 10^{-4}$ for the treatment group and $- 5.4 \times 10^{-4}$ for the control group), we infer that as the education level of treatment group users increases, the severity of the effect of sharing misinformation on their anxiety decreases. To analyze this trend more closely, we dropped the outliers from the current analysis by only considering users with increase in post-treatment/placebo anxiety and ARI score that fall within two standard deviations around respective mean values (i.e., $\mu \pm 2\sigma$). As shown in inset of Fig. 2 B, we find the same trends with tighter confidence intervals. Specifically, the slope for the treatment group users is $-2.9 \times 10^{-4}$ (95% CI: $[-6.3 \times 10^{-4}, 7.5 \times 10^{-5}]$) and for the control group users is $-1.9 \times 10^{-4}$ (95% CI: $[-5.1 \times 10^{-4}, 8.4 \times 10^{-5}]$).

## Discussion

### Interpretation of results

Our study identifies users who are similar in terms of a wide range of attributes, including prior-anxiety levels, and demonstrates that the ones who share misinformation experience exacerbated anxiety. Previous research highlights the positive role of prior anxiety in determining the tendency to believe in rumors and further share them^30–32^. While these studies have found that prior anxiety drives individuals to forage for information, making them susceptible to misinformation, they did not study how exposure to misinformation affects their consequent mental health. A positive causal effect of sharing misinformation on anxiety, as observed in our work, likely indicates a vicious cycle where highly anxious individuals are more vulnerable to believing and sharing misinformation and ${\text {consequently may experience exacerbated}}$ anxiety by sharing misinformation, thereby increasing their vulnerability towards future misinformation. The implications of the existence of this vicious cycle are threatening, as it can trigger anxiety disorders in those without an existing psychiatric morbidity^13,14^ and can even lead to suicidal ideation among those with existing conditions^15,16^. More specifically, the existence of this vicious cycle may provide empirical evidence in support of the social amplification theory^62^, wherein the risks of crises, like the COVID-19 infodemic, are first amplified during the spread of (mis)information, triggering behavior that in turn, leads to further amplification of the risks^63,64^. Future investigations can aim to study this cyclic effect of (a) anxiety on exposure to misinformation (as indicated by prior investigations), and (b) exposure to misinformation on anxiety (as indicated by our research), in a single study.

While there have been active discussions on why people share misinformation on social media^29,34^, it is worth noting that sharing misinformation, albeit conservative, is a strong and robust signal of being exposed to misinformation^35,36^. Our study demonstrates that regardless of the underlying cognition or intent behind sharing misinformation, it adversely affects the anxiety of those who share misinformation on social media. Our findings also pave the way for future research that could explain the psychological reasons or the mechanisms behind the causal relationship between sharing misinformation and experiencing exacerbated anxiety.

Our socio-demographic analysis indicates that women and racial minorities in the U.S. experience more anxiety as a consequence of sharing misinformation than men and whites, respectively. These results highlight the disparities in experiencing the indirect effect of the pandemic and add to the existing reports and articles that argue how the pandemic has disproportionately impacted women^65^ and racial minorities^66^. We also find that higher education level subdues the effect of sharing misinformation on anxiety, pointing to the potential of pandemic and health literacy initiatives. This effect of education level on anxiety experienced in response to exposure to misinformation adds to the existing body of research in psychology that indicates that people demonstrating lower cognitive abilities are more affected by false information^67^, even after being presented with corrected information^68^.

### Theoretical and practical implications

The findings from our study can enable informed decision-making by policy-makers who are responsible for regulating misinformation and monitoring mental health. The negative impact of misinformation on the anxiety of vulnerable individuals can influence the decisions concerning resource allocation and prioritization while moderating its spread in a setting where fact-checking is severely resource-constrained^69,70^. Additionally, agencies responsible for mental well-being can devise strategies to provide timely and proactive care to protected groups in events of frequent exposure to misinformation during an ongoing and protracted crisis like COVID-19.

Beyond the theoretical implications, we believe our methods demonstrate the crucial role that social media data and machine learning techniques can play during global public health emergencies. Our methods can be used to build tools that could allow social media platforms to assess the relative increase in people’s anxiety post sharing of misinformation and alert them or appropriate mental health caregivers or interventions sufficiently in advance. The need for such directed interventions is further underscored by the fact that the First Amendment protections in the U.S. present significant challenges for regulatory remedies that aim to moderate the spread of misinformation on social media, via approaches like deplatforming, banning, or content removal, all of which have been major sources of controversy recently^71^. As a potential targeted intervention, social media platforms can algorithmically adapt the personalized feeds of at-risk individuals to limit their exposure to misinformation, and in turn, to misinformation sharing and the consequent anxiety.

Some social media corporations have shown increasing commitment to maintaining the “health” of conversations that unfold on their sites, and identify means to promote healthy conversations^72^. Our findings can add new evidence to the efforts of social media corporations, potentially augmenting existing efforts and interventions that seek to protect the mental health of vulnerable individuals and subgroups, as well as to realize the vision of Jürgen Habermas’ public sphere online^73^. We believe these interventions can work complementary to or in concert with relentless efforts and campaigns of public health organizations encouraging individuals to fact-check pandemic related information before sharing or believing them, such that resulting emotional trauma may be minimized. While the exact nature of the interventions constitutes ripe areas of future work, they could take the shape of advice and pointers to reduce the duration and frequency of social media consumption, careful selection of authentic and scientific online sources of COVID-19 related information, practicing healthy and alternate coping techniques for stress (e.g. mindfulness), and improvement in lifestyle behaviors such as sleep hygiene and exercise routines.

### Limitations and future work

It is also important to be clear about the limitations of this study. First, we conducted an observational study instead of an RCT. It is worth noting that even though observational studies offer several complementary advantages over RCTs—for instance, greater statistical power and generalizability^40^, they cannot account for unobserved confounding. However, our causal inference framework adopted a matching-based approach that simulated an RCT by controlling as many covariates as possible, reducing the effect of unobserved confounding^52^. Second, it can be argued that machine learning classifiers do not infer misinformation and anxiety levels with perfect accuracy and this can lead to accumulation of errors in the overall causal inference framework. However, automated detection of misinformation and inference of anxiety allows for unobtrusive behavioral sensing of a wider range of subjects than using traditional surveys or questionnaires. We discuss the methodological gaps that should be considered while deriving proxy signals from social media data to infer elements of mental health, such as anxiety of individuals, in Supplementary Information, Sect. 7.2. Third, we note that an automated way to gauge demographic attributes in shared content is considered an ethically thorny issue^74^ as it might call upon negative impacts such as social discrimination and rejection, or even exacerbate some of the very stressors considered here, such as anxiety, that are detrimental to well-being. We caution that our findings are not suitable to be and should not be adopted as a standalone mechanism to connect anxious social media users holding specific racial or gender identities with mental health care. Instead, due to a reliance on automatically inferred socio-demographics, our findings are best used as part of an ecology of evidence-based approaches to address the harms of online misinformation. Further, for our socio-demographic analysis, we considered only binary sex (men/women) and only the four major races in the U.S. (white, Black, Asian Pacific Islander, and Hispanic). Importantly, the state-of-the-art demographic inference methods that we adopted for our analysis exclude certain marginalized communities or even erase certain identities, for instance, LGBTQ+ or mixed race identities. While the current study highlights the disparities among certain demographic groups, we believe that our analysis will need to be extended to include additional minority identities in the future and we advocate the use of an intersectional approach^75^ to tackling the mental health challenges of online misinformation. We elaborate on these limitations in Supplementary Information, Sect. 7.

In future research, we intend to expand beyond sharing as the type of exposure signal that we consider to include weaker exposure signals such as ‘liking’ a misinformative post or commenting on it. Such exposure signals should be contextualized appropriately—for instance, the act of commenting cannot be isolated from what the comment is about; whether it favors, counters, or is neutral to the misinformative post. It may be valuable to categorize misinformation into various types such as health-related and conspiratorial political misinformation, to understand how exposures to different types of misinformation affect anxiety. Additionally, we also intend to expand our study to understand the impact of exposure to misinformation on other mental health indicators beyond anxiety, like depression and stress.

## Conclusion

In sum, we have shed light on the causal effect of sharing misinformation on experiencing exacerbated anxiety and the disparities that exist in experiencing this adverse effect across different socio-demographic groups. Given the massive number of people who are exposed to misinformation that accompanies almost every major real-world event, our study indicates that the people who share misinformation do not just worsen the situation by contributing to its spread, as is known widely, but are also the victims of resultant anxiety that they experience. Directing appropriate resources to the vulnerable groups, both algorithmically and through caregivers, can help in mitigating the adverse effects of misinformation on anxiety.

## Supplementary Information

Supplementary Information.

## Data Availability

Abiding by the Terms of Service of Twitter, upon acceptance, we will make available, the IDs of all the users in our study, and appropriate labels for each of these users—count of COVID-19 misinformation posts shared by the users, which users were assigned to the treatment and control groups, their socio-demographic labels (sex, race, and ARI score), and prior and post-treatment/placebo anxiety scores. We will also disseminate the algorithmic artifacts from this study into a readily usable toolkit; the data and toolkit will be made available via an easily accessible platform like GitHub, with appropriate documentation.
