# Supplementary material for: Examining the impact of sharing COVID-19 misinformation online on mental health
Source: Sci Rep. 2022 May 16;12:8045. doi: 10.1038/s41598-022-11488-y (PMC9109204; doi:10.1038/s41598-022-11488-y)
Supplement: Supplementary file 1 — Supplementary Information. [file 41598_2022_11488_MOESM1_ESM.pdf]

Examining the Impact of Sharing COVID-19 Misinformation Online on Mental Health

Supplementary Information

Gaurav Verma, **Ankur Bhardwaj**, **Talayeh Aledavood**,
Munmun De Choudhury, and **Srijan Kumar**

Contents

1 Data Collection Procedure and Statistics **2**

2 Methods **2**
2.1 Misinformation Classifier . . . 2
2.2 Anxiety Scorer . . . 3
2.3 Assignment of Users to Control and Treatment Groups . . . 3
2.4 Assignment of Treatment and Placebo Dates . . . 4
2.5 Causal Inference Approach . . . 4
2.6 Matching Users Across the Two Groups . . . 4
2.7 Quality of Matching . . . 6
2.8 Treatment Effect Estimation . . . 6

3 Results **7**
3.1 Demographic Analysis . . . 7

4 Sensitivity to Design Choices **8**

5 Effect of Bot Accounts **8**

6 Validity of SUTVA **9**

7 Limitations **9**
7.1 Observational Study Versus Randomized Controlled Trial . . . 9
7.2 Using Machine Learning to Identify Misinformative Twitter Posts and Infer Anxiety 9
7.3 Inferring Demographic Attributes . . . 10

Figures S1 to S5 **11**

Tables S1 to S3 **16**

1 Data Collection Procedure and Statistics

The dataset for this study was created using the Twitter API [1]. We started our data collection by collecting Twitter posts on COVID-19. Specifically, we collected the posts that contained at least one of the following keywords or their variants: 'covid', 'corona', 'sars-cov-2', 'coronavirus', and 'covid19'. We also collected all the Twitter posts in the timelines (from Jan 1, 2019, to July 15, 2020) of the users who made these posts.

To handle outliers in our data, we removed verified Twitter accounts, users with zero followers or followees, or those with followers (or followees) greater than two times the standard deviation added to the mean number of followers (or followees) ($\mu+2\sigma$). Additionally, to ensure consistency in our data, we dropped the accounts that were created after January 1, 2019, from our analysis. We also removed users who shared less than 10 posts in each of the pre-COVID-19 (January 1, 2019 — December 30, 2019) and post-COVID-19 (December 31, 2019 — July 15, 2020) periods of our analysis. The descriptive statistics of the filtered dataset is given in Table S1.

The two variables of interest in this study are misinformation and anxiety. We describe the approaches that we use to infer the misinformativeness of all Twitter posts in our dataset and their anxiety levels in the Section 2.1 and 2.2, respectively.

2 Methods

2.1 Misinformation Classifier

For all the users in our dataset, towards the research goal of this work, we devised an approach to identify if their posts contained COVID-19 related misinformation or not. Machine learning and natural language processing offer classification tools that allow unobtrusive and large-scale detection of COVID-19 related misinformation in Twitter posts. We built a text-based misinformation classifier using an effective transfer learning technique – Universal Language Model Fine-Tuning (ULMFiT) [2]. We chose ULMFiT because it has shown remarkable performance on several classification tasks in natural language processing (NLP) with a limited amount of labeled data [2]. Figure S1 gives an overview of the training strategy that we adopted to train the misinformation classifier.

Training the ULMFiT based misinformation classifier involves two steps: (a) The first step is to fine-tune the weights of a pre-trained general domain language model using COVID-19 related unlabeled Twitter data, and (b) the second step involves training the encoder of the hence obtained language model on the classification task using labeled Twitter posts. Here, the first step helps in training a specialized language model as it ensures that the fine-tuned language model is aware of the vocabulary and linguistic structure surrounding the topic of COVID-19, whereas the second step further adds additional linear layers on top of an encoder and trains the model to classify text as either misinformative or not.

We started with a language model (LM) based on the standard Average Stochastic Gradient Descent (A-SGD) Weight-Dropped Long Short Term Memory (AWD-LSTM) architecture [3], which is pre-trained on Wikitext-103 dataset. The pre-training task of this language model is to predict the next word given a sliding window of few previous words. This gives the model a general ability to model the English language in a self-supervised manner. To further adapt this language model to incorporate linguistic aspects that are specific to COVID-19, we fine-tuned it to predict the next words in 120,000 Twitter posts related to COVID-19. Since the ULMFiT architecture consists of multiple LSTM layers that capture different types of linguistic information [2], we used the discriminative fine-tuning approach [2] to set different learning rates for each layer in the architecture. After unsupervised fine-tuning of the LM, we used its encoder to train a misinformation classifier. We further fine-tuned the encoder on a binary

classification task (misinformative-or-not) using a COVID-19 misinformation posts dataset that comprises 12,839 labeled Twitter posts [4]. Micallef et al. [4] manually labeled these 12,839 Twitter posts based on whether they contain COVID-19 related misinformation spanning across 6 different categories (enumerated in Table S2) or not — 9,478 posts were labeled negatively (i.e., no misinformation) and 3,361 posts were labeled positively. As per Howard et al. [2], we used gradual unfreezing while fine-tuning the encoder for the binary classification task. The underlying idea is to gradually unfreeze the layers starting from the last layers (i.e., closest to the softmax output) as it contains the least general knowledge.

For training and evaluating the misinformation classifier, we divided the unbalanced dataset of 12,839 labeled Twitter posts in a 70 : 30 ratio for training and validation, respectively. Using the ULMFiT approach described above, we trained the classifier and evaluated its performance using precision and recall values on the validation set. To ensure higher confidence in assigning users who share misinformation to the treatment group, we analyzed the trade-off between precision and recall on the validation set with varying prediction probabilities, as shown in Figure S2. Finally, we chose the threshold classification probability of 0.70 as it provides high enough precision of 0.90 in identifying misinformative Twitter posts. At 0.70 threshold, the recall of the model was 0.66; F_1 score = 0.76; and accuracy = 0.89.

2.2 Anxiety Scorer

Social media platforms like Twitter provide a means for capturing behavioral attributes that are relevant to an individual's thoughts and mood – the emotion and language used in Twitter posts can be used to infer feelings of stress and anxiety [5, 6, 7, 8]. To this end, we used the classifier developed by Saha et al. [8] to score the anxiety level of Twitter posts on a scale of 0 to 1 using the predicted class probabilities.

This anxiety scorer is a support vector machine (SVM) based classifier with linear kernels that uses 5,000 n-grams (where, $n = 1, 2, 3$) as features. We used the binary classifier trained on Reddit data, where the positive examples came from posts on `r/anxiety` community on Reddit and the negative examples came from other communities such as `r/AskReddit`, `r/aww`, and `r/movies`. Recent work has demonstrated that even though the anxiety classifier was originally trained on Reddit data, its classification performance transfers reasonably well to Twitter data, presenting an accuracy of about 90% on held-out test data from Twitter [9]. In Table S3, we present some randomly sampled Twitter posts with anxiety scores in different ranges, to qualitatively illustrate the inference capabilities of the anxiety scorer.

2.3 Assignment of Users to Control and Treatment Groups

Our causal inference framework requires categorizing Twitter users into two groups — those who shared misinformation (treatment group) and those who did not (control group). We used the misinformation classifier to classify each post in all the user timelines as either misinformative or not. We show the histogram depicting the frequency of users for number of misinformative posts they share in Figure S3. Based on this distribution, we assigned all the users who shared at least five COVID-19 misinformative posts between Jan 1, 2020, and July 15, 2020, to the treatment group. Since our causal inference approach only allows for binary treatment values (either present or absent), it is imperative that we keep a well-defined boundary of separation between treatment and control groups. We assigned all the users with 0 misinformative posts to the control group, and dropped all the other users who shared non-zero but strictly less than five misinformative posts from our analysis. Dropping these users ensured that the treatment group and control group are sufficiently distinct from each other in terms of misinformation sharing

behavior. Using this assignment technique, we find that out of the 43,832 users, 1,288 users were assigned to the treatment group and 31,002 users were assigned to the control group.

2.4 Assignment of Treatment and Placebo Dates

This study involves measuring the change in anxiety after misinformation has been shared by a Twitter user. We consider the date when a user shared their first COVID-19 related misinformation as their treatment date. However, since the users in the control group never shared COVID-19 related misinformation in the duration of the analysis, we assigned them a placebo date by matching the non-parametric distribution of treatment dates. Similar distributions of placebo and treatment dates mitigate the effect of any temporal confounds. For this, we non-parametrically simulated placebo dates from the pool of treatment dates and measured the similarity in their distribution using Kolmogorov-Smirnov test. We obtained an extremely low statistic of 0.03, indicating a similarity in the probability distribution of treatment and placebo dates (Figure S4). The treatment and placebo dates helped in dividing the timelines of users in treatment and control groups, respectively, into before and after treatment/placebo segments. It is worth noting that since our matching procedure, as discussed in Section 2.6, requires that the behavioral indicators prior to treatment/placebo are similar for users within a stratum, the assignment of treatment and placebo dates can only be done before matching the users based on their propensity scores. However, to ensure that the distribution of treatment and placebo dates are similar within each matched stratum (along the overall similarity of distributions, as demonstrated in Figure S4), we compute the post-matching K-S statistic of each valid stratum and report the statistics in Section 2.7.

Besides the individual-specific treatment or placebo dates, we note that the first misinformation post across all the users in our dataset was shared on January 21, 2020; we consider this date as the global first treatment date.

2.5 Causal Inference Approach

The causal inference approach that we adopted for this study is based on the potential outcomes framework [10, 11]. Potential outcomes framework compares two potential outcomes: 1) $Y_i(T = 1)$ when a user is exposed to treatment T, and 2) $Y_i(T = 0)$ when a user is not exposed to T. Since in an observational study it is impossible to find individuals who have both the outcomes (as a single user cannot be both exposed and not exposed to a treatment), we estimated the missing counterfactual outcome for an individual based on the outcomes of other similar (matched) individuals. For this, we employed stratified propensity score analysis [12] to first match the users and then compared the outcomes of matched users across the treatment and control groups.

It is worth mentioning that the adopted causal inference approach necessitates considering the treatment (i.e., sharing misinformation) as a binary variable (either present or completely absent). However, there is no such constraint on the outcome variable (i.e., consequent anxiety) and therefore, we considered anxiety to be a continuous variable. This consideration of anxiety as a continuous variable allows for a finer analysis of the effect of treatment on the outcome, which would not have been the case if we considered anxiety as a binary variable as well due to resulting loss of information.

2.6 Matching Users Across the Two Groups

Here we discuss our two-level strategy to identify and match similar users across the control and treatment groups. To provide a quick overview of our approach, we first stratified all the users

based on their propensity scores – the likelihood of receiving treatment, and then matched users from the two groups within each stratum based on the similarity of linguistic patterns observed in their Twitter timelines.

The stratified propensity score matching technique attempts to distinguish the effects of specific treatment (i.e., sharing misinformation) from the effects of covariates (number of followers/followees, Twitter activity, prior anxiety, etc.) by dividing the treatment group and the control group into strata such that the covariates of the treatment subgroup within a stratum are statistically similar to the covariates of the control subgroup within the same stratum. This stratification of users is based on their estimated propensity to receive treatment. The estimated propensity is a machine-learned function of a user's likelihood of receiving a treatment based on the user's covariates.

We trained a logistic regression classifier to implement the propensity score function using a range of covariates from pre-treatment user timeline on Twitter (i.e., from January 1, 2019 to the user-specific treatment date) as features. By considering different types of covariates as features for the logistic regression model, we were able to control for platform-specific behavioral attributes as well as pre-treatment mental health indicators including prior anxiety. Since the binary classification task involves predicting the status of treatment using the covariates, where 1 corresponds to the presence of treatment while 0 corresponds to its absence, the prediction probability of class 1 estimates the propensity score. Inspired from prior work in causal inference on social media platforms [5, 8], we used four different types of covariates as features: (i) Twitter account meta-information, namely number of followers/followees, and account creation date; (ii) timeline information namely, total number posts, weekly post rate, monthly post rate, average likes, and average retweets; (iii) psycholinguistic aspects related to attentional focus, emotionality, social relationships, thinking styles, and individual differences displayed in posts (using the LIWC dictionary) [13, 14]; and (iv) pre-treatment mental health indicators, capturing the levels of depression, anxiety, stress, and psychosis. We used the scorers built by Saha et al. [8] to quantify the four pre-treatment mental health indicators. To represent a user's prior mental state across these dimensions, we created a frequency distribution vector using 10 equally-spaced buckets such that the first bucket contained the number of posts with anxiety score $\in [0, 0.1)$ in their pre-treatment period and so on.

Figure S5 shows the distribution of propensity scores estimated using the logistic regression classifier discussed above, for both the treatment and control group users. We constructed 50 equally-spaced strata $\in [0, 1]$ and assigned users from both the control and treatment groups to these strata based on their estimated propensity scores. Since the covariates of users in the treatment subgroup within a stratum are statistically identical to the covariates of users in the control subgroup of that stratum, each stratum simulates a randomized controlled trial where the assignment of treatment is uncorrelated with covariates. To ensure greater linguistic similarity between control and treatment group users of each stratum, we performed another post-stratification round of matching [8]. For each stratum, we dropped the control group users who had an Intersection over Union (Jaccard index) of less than 0.33 between their top-1000 unigrams and those of any of the treatment group users within the stratum. We explored different threshold values and chose 0.33 as it provides a reasonable trade-off between strength of matching (higher threshold would lead to a stricter matching criterion) and resultant population of the stratum (higher population would lead to greater statistical strength); see Section 4 for more details. The top-1000 unigrams are obtained from all the posts in the pre-treatment/placebo timelines of users. Following this, we dropped those strata that do not have enough support in terms of the number of treatment or control group users from our analysis, as is typical in causal inference research [6]. We quantify the quality of matching in the next section.

2.7 Quality of Matching

To ensure that our two-level matching strategy indeed matches statistically comparable treatment and control group users, we evaluated the balance of their covariates by computing the standardized mean difference (SMD) across covariates in the two groups in each valid stratum. Mathematically, SMD expresses the difference in the mean covariate values of the two groups as a fraction of the pooled standard deviation of the two groups. As per Kıcıman et al. [15], an SMD lower than 0.2 indicates that the covariates of the two groups are balanced and that the adopted matching strategies are effective. We note that all valid strata had an SMD strictly less than 0.2, and the mean SMD was 0.011 (maximum = 0.073).

To assess whether the distributions of within-stratum treatment and placebo dates are similar, we computed the K-S statistic of all the valid stratum. We find that all the strata of matched users have a low K-S statistic (< 0.1), with mean K-S statistic of 0.07, indicating that the treatment and placebo dates within each stratum are drawn from the same underlying distribution.

2.8 Treatment Effect Estimation

The final step in our causal study was to estimate the effect of sharing misinformation (i.e., the treatment) on the anxiety levels of individuals who demonstrate such behavior (i.e., the outcome). For this, we quantified the treatment effect (TE) within each stratum as the difference of increase in post-treatment anxiety levels with respect to the pre-treatment anxiety levels between treatment and control groups. Mathematically, for stratum i,

$$TE_i^{abs} = (A_{after}^{trt} - A_{before}^{trt}) - (A_{after}^{ctrl} - A_{before}^{ctrl}). \quad (1)$$

Here, A^{trt} denotes the average anxiety of all treatment group users in stratum i, and is measured by taking the three-month average of anxiety scores corresponding to all posts from the users. Similarly, A^{ctrl} denotes the average anxiety of all control group users in stratum i. Depending on the subscript in Equation 1, we either considered the three-month period after the treatment or placebo date or before the global first treatment date. Equation 1 essentially quantifies the absolute additional increase in anxiety scores of treatment group users with respect to the increase in anxiety scores of control group users. It is worth noting that for the users in the treatment group, we did not take the posts that were identified as misinformative while computing the average anxiety scores. This ensured that the behavioral indicators that were used to assign users to treatment and control groups did not contribute toward the quantification of the outcome. Next, we took a weighted average of treatment effect across strata to obtain the mean treatment effect of sharing misinformation on anxiety. To further interpret the effect of sharing misinformation on anxiety, we also computed the treatment effect using the following equation, which provides an estimate for the relative additional increase in anxiety among treatment group users with respect to the increase in anxiety among control group users. It is worth noting that for any stratum i in our dataset, A_{after}^{ctrl} is always greater than A_{before}^{ctrl}, which effectively ensures that the denominator in Equation 2 is always positive. As a side note, $A_{after}^{ctrl} > A_{before}^{ctrl}$ implies that control group users, in general, experienced an increase in anxiety during the COVID-19 pandemic — an observation that is also supported by other studies that analyze the psychosocial impact of COVID-19 [16, 17].

$$TE_i^{rel} = \frac{(A_{after}^{trt} - A_{before}^{trt}) - (A_{after}^{ctrl} - A_{before}^{ctrl})}{A_{after}^{ctrl} - A_{before}^{ctrl}}. \quad (2)$$

3 Results

We observe a positive effect of sharing misinformation on anxiety, indicated by an absolute treatment effect of 0.063 and a relative treatment effect of 2.011. To further support our main findings around the causal effect of sharing misinformation on experiencing anxiety, we computed stratum-level Cohen's d between the distributions of post-treatment anxiety and post-placebo anxiety. We find that the average of Cohen's d for all strata is 0.59, indicating a medium to large effect size. A Cohen's d of less than 0.2 indicates a trivial difference that can be ignored. However, in our analysis, we observe that all valid strata have a Cohen's d > 0.2. Furthermore, an unequal variances (Welch's) t-test on distributions of post-treatment and post-placebo anxiety outcomes revealed that the effect is statistically significant ($t < [-0.31, 7.47]; P < .01$). In conjunction with the treatment effect estimates, these findings further reinforce the presence of a causal relationship between sharing misinformation and experiencing exacerbated anxiety.

3.1 Demographic Analysis

We first identified a subset of treatment group users who were located within the United States using the geo-code in their Twitter posts and the self-described location in their Twitter description field (N = 762). For comparison with control group users, we also identified a subset of control group users who were located within the U.S. (N = 1198). We compared the first names of these users against the database of names since 1880, as reported by the U.S. Social Security Administration, to find the most probable sex. Similarly, we compared the last names of individuals against the database of names per the 2010 U.S. census data to identify the most probable race. We then manually browsed through the Twitter description of all the users and updated these automated inferences based on explicit self-reports of sex or race (for instance, mom, mother, wife, daughter indicates that the user is a woman and dad, father, husband, son indicates that the user is a man). These methods have been used to accurately infer demographics attributes in prior in social computing studies [18, 19, 20, 21, 22, 23, 16].

We find that among the treatment group users within the U.S. (N = 762), the percentage of men is 5.94% higher than the percentage of men in the control group (N = 1189). However, the percentage of women is 5.68% lesser in the treatment group when compared to the control group. Additionally, whites have 9.20% additional representation in the treatment group than in the control group, whereas Blacks, APIs, and Hispanics have lesser representation in the treatment group. This indicates a higher tendency among men and whites to share COVID-19 related misinformation. The complete distribution of control and treatment group users across these demographic dimensions is shown in Table S4.

For the purposes of inferring education level using Twitter posts, we used the Automated Readability Index, as has been done in several prior studies [24, 25, 26]. We did not consider any retweets while computing the ARI for a user. This was done to ensure that only originally authored posts are considered to infer a user's education level. The line of best fit for variation in post-treatment anxiety of treatment groups users with ARI of has a slope of -7.1×10^{-4} and a y-axis intercept of 0.034. For treatment group users, OLS regression between the two variables yields an R-squared of 0.52. For control group users, the line of best fit has a slope of -5.4×10^{-4}, y-axis intercept of 0.024, and the OLS regression yields an R-squared of 0.46. Besides using ARI scores for inferring education level, we also used other stats like Flesch-Kincaid readability scores [27] and found similar results as presented in the main manuscript under 'Education Level'. Using Flesch-Kincaid readability score instead of ARI yielded a line of best fit with slope -6.4×10^{-4} and y-axis intercept 0.031 for the treatment group and slope $= -5.1 \times 10^{-4}$ and y-axis intercept = 0.022 for the control group.

The p-values reported for demographic analyses in the main text were calculated using a two-sample t-test with equal variances assumption.

4 Sensitivity to Design Choices

We re-ran our experiments while varying two crucial hyper-parameters – the minimum number of misinformative posts shared by a user for them to be assigned to the treatment group, and the threshold Intersection over Union (Jaccard index) between matched users across treatment and control groups. We note that the positive effect of sharing misinformation on anxiety is robust against these variations, and the effect size and statistical significance of our results persist. As we increased the threshold number of misinformative posts from its original value of 5, the treatment effect (TE^{rel} as well as TE^{abs}) increased. Similarly, a decrease in TE is observed with smaller values of minimum misinformative posts.

Increasing the threshold of shared misinformative posts to 7 (from original value of 5) resulted in a TE^{rel} of 4.639 ($P < .01$), while decreasing it to 3 (from 5) resulted in TE^{rel} of 1.082 ($P < .01$). These observations can be attributed to the increased (or reduced) separation in behaviors of the control and treatment group users as the minimum number of misinformative posts shared by treatment group users was increased (or decreased).

Since increasing the Jaccard index implies a stricter matching criterion, the population of each stratum and the overall number of consequent valid strata decrease. This leads to lesser statistical strength of the results. To arrive at the optimal value of the threshold Jaccard index, we set the value of Jaccard index to $\frac{1}{n}$ $\forall n \in \{2, 3, \ldots 10\}$ (sweeping the values based on n^{-1} is more effective in this case than setting fixed intervals because the number of users who pass the matching criteria drops severely as the minimum Jaccard index increases), and observed the mean SMD of resultant strata to assess the quality of matching. Setting the minimum Jaccard index to 0.33 (n=3) yielded strata where all the strata-level SMD values were < 0.2, indicating a good match of users across the treatment and control subgroups [15] along with a sufficient number of strata as well as population in each stratum ($>= 20$ valid strata and $>= 40$ matched users in each stratum). For $n \in \{4, 5, 6, 7, 8, 9, 10\}$, we found at least one stratum to have the SMD value as > 0.2. Since this indicated a poor quality of matching, we did not select the threshold Jaccard index based on any of these values of n. For $n = 2$ the number of valid strata dropped to 7 with < 10 matched users in most of the strata (severely limited statistical strength due to small sample size). To this end, we set $n = 3$ and threshold Jaccard index to be 0.33 for the second stage of matching as it provides the right balance between quality of matching and sample size.

5 Effect of Bot Accounts

Here we discuss the presence of bot accounts in our study. We used the Botometer API [28] to assign a bot score to all the 32,290 Twitter accounts in the treatment and control groups. Following previous studies [29, 30], we used a threshold of 0.5 to classify an account as a bot. We find that only 1.66% of all the users are classified as bots (1.71% of treatment group users and 1.65% of control group users). However, considering the shortcomings of Botometer, as highlighted by [31], we also used the DeBot system [32, 33] to double-check the bot status of all accounts. We find that out of the 32,290 users in our analysis, only 96 ($\sim$ 0.3%) occur in the list of bot accounts maintained by DeBot. The extremely low fraction of bot accounts can be attributed to a combination of filtering criteria followed in this study. Thus, our analysis is focused on analyzing humans.

6 Validity of SUTVA

The 'no interference' part of Stable Unit Treatment Values Assumption (SUTVA) [34, 12] requires that the potential outcomes (here the outcome is anxiety) for any unit (Twitter users) do not vary with the treatments (sharing misinformation) assigned to other units (users). In the context of this study, however, it is possible that the users within the control group may experience anxiety because of being exposed to misinformation that their followees may have shared. The presence of such a phenomenon where control group users experience anxiety due to shared misinformation, albeit by others, would indicate a higher treatment effect than what we observe in our analysis – further supporting the argument in favor of the observed positive effect of sharing misinformation on anxiety. To empirically establish the validity of SUTVA, we randomly sampled 20 users from our control group and analyzed the Twitter posts of their 6,137 unique followees. We found that out of the 2 million posts analyzed, only 41 posts were classified as misinformative by our classifier. Furthermore, out of the 6,137 unique followees, only 2 users had shared more than 5 misinformative posts in the entire duration under consideration. This is an expected observation as prior works have indicated that misinformative posts are largely shared within 'echo chambers' [35, 36]. This analysis indicates the validity of SUTVA in our context.

7 Limitations

7.1 Observational Study Versus Randomized Controlled Trial

As mentioned in the main manuscript, a randomized controlled trial in our context would present ethical challenges. While observational studies present a strong alternative to RCTs, it is worth noting that they cannot account for unobserved confounding. However, observational studies offer several complementary advantages – for instance, greater statistical power and generalizablity over RCTs, since RCTs often only employ a handful of subjects (N $\sim$ 100) with a restrictive sampling criteria [37]. Furthermore, our causal inference framework adopts a matching-based approach that simulates an RCT by controlling for as many covariates as possible, reducing the effect of unobserved confounders [10].

7.2 Using Machine Learning to Identify Misinformative Twitter Posts and Infer Anxiety

We trained two classifiers to identify misinformative posts and infer the anxiety level. It can be argued that machine learning classifiers are not completely accurate and this can lead to accumulation of errors in the overall causal inference framework. However, quantitative and qualitative evaluations of both the classifiers suggest their efficacy in automated identification and inference of misinformation and anxiety scores, respectively. Furthermore, automated detection of misinformation and inference of anxiety score presents a few major advantages over collecting similar information using surveys and questionnaires. Using these classifiers allowed us to work with a large number of users, with a greater coverage within the sample than what surveys/questionnaires would have allowed, and consequently strengthened our observations. Automated identification and inference also allow for unobtrusive behavioral sensing of subjects in the study. It is worth noting that using predictive models to infer the mental health outcomes from social media data, such as using the anxiety levels of an individual's Twitter posts as a proxy of their mental health, is prone to uncertainty in the construct validity of proxy signals – wherein a substantive difference has been observed in the internal validity of these inference methods and their external validity on unseen patient test data [38].

7.3 Inferring Demographic Attributes

This work considers only binary sex (men/women) and only the four major races in the U.S. (white, Black, Asian Pacific Islander, and Hispanic). The state-of-the-art demographic attribute inference methods that this study relies on are severely limited in this sense. It can be argued that our work is non-inclusive of certain marginalized communities. While the current study highlights the disparities among certain demographic groups, we believe that our analysis can be extended to include more minority identities in the future.

Figures S1 to S5

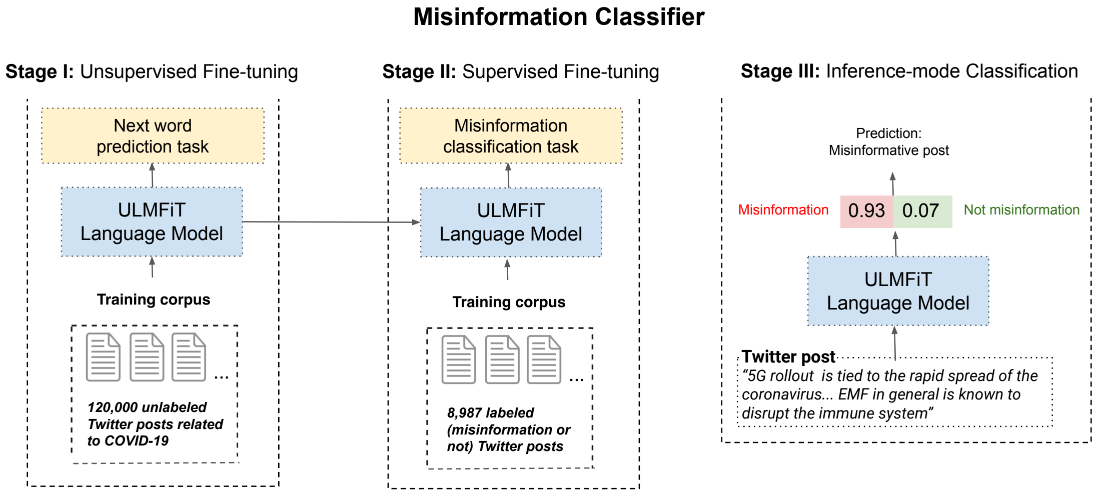

Figure 1: The two-stage training strategy adopted to train the misinformation classifier. The first stage involves unsupervised fine-tuning to adapt the language model's parameters to incorporate COVID-19 related vocabulary and linguistic structure. The second stage involves supervised fine-tuning to identify whether a given Twitter post contains COVID-19 related misinformation. Once trained, the model is used in inference mode to identify misinformative posts in the dataset.

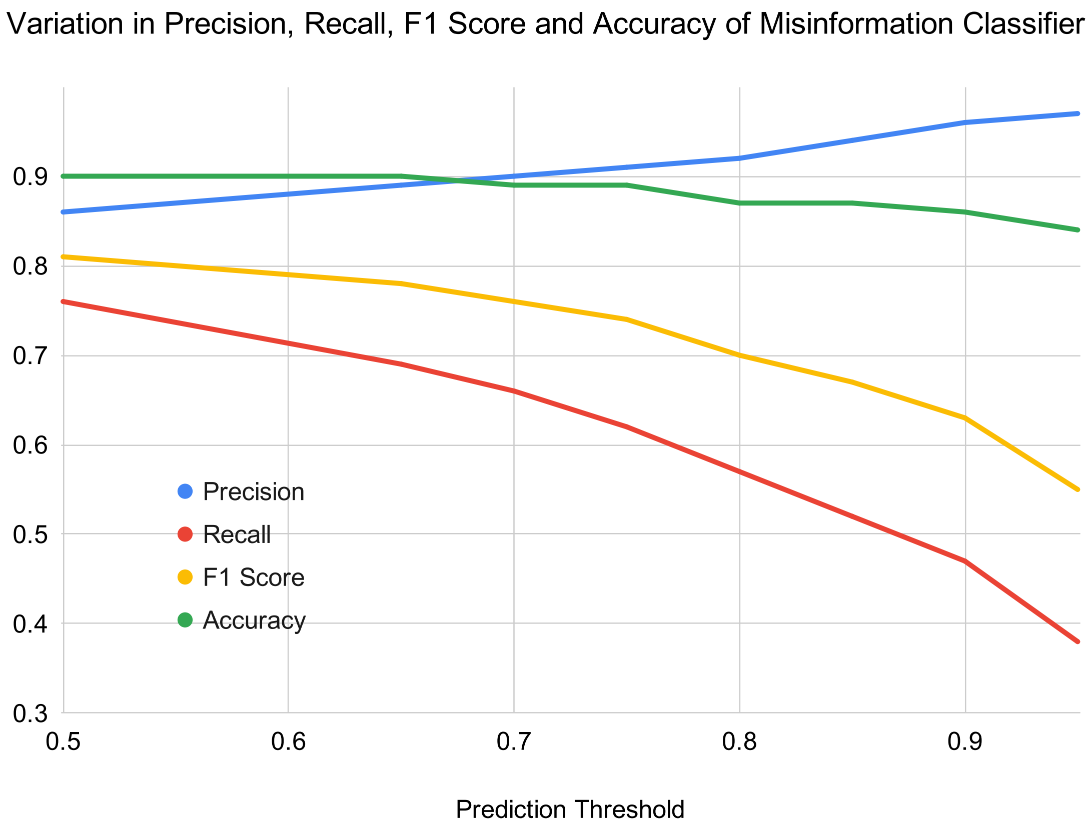

Figure 2: The variation in the performance metrics of the trained misinformation classifier with different values of prediction threshold.

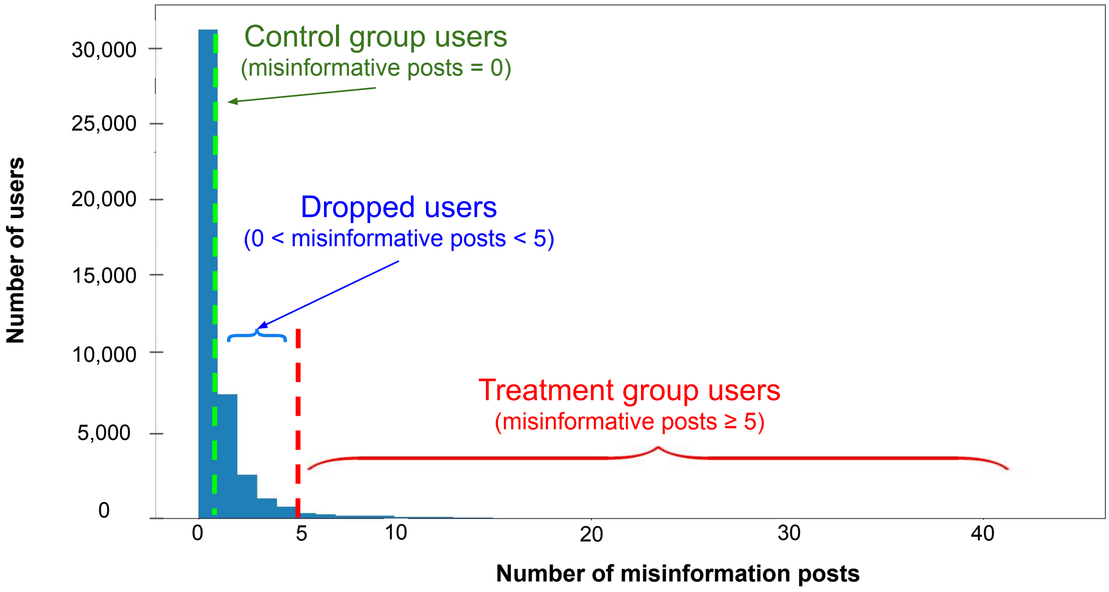

Figure 3: Frequency of users for number of COVID-19 related misinformative posts.

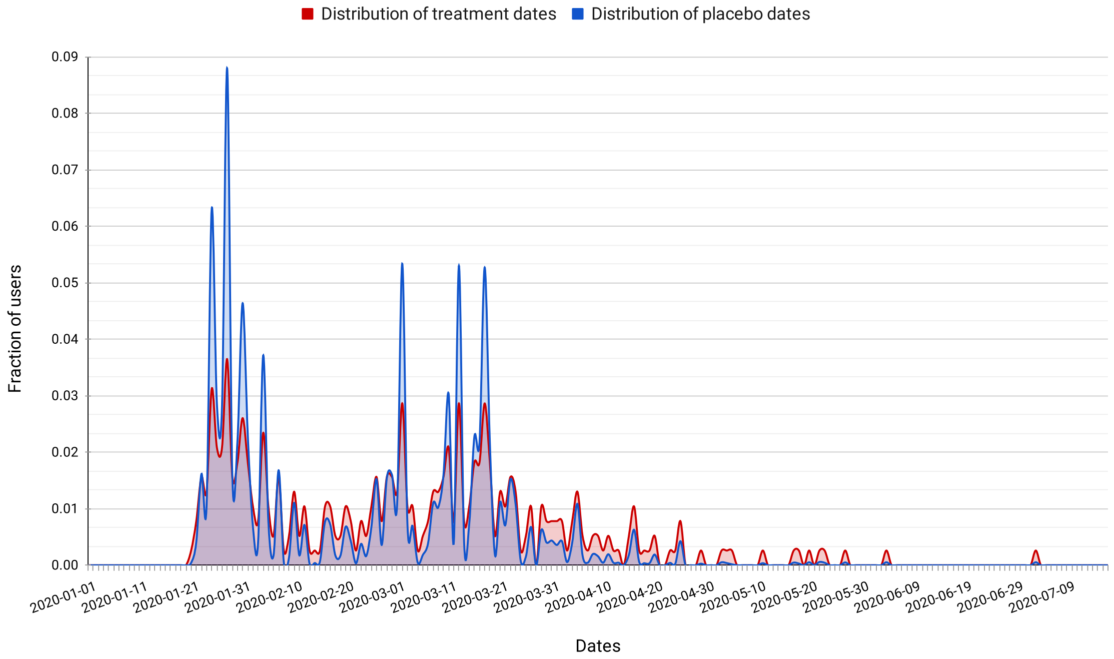

Figure 4: Distribution of treatment and placebo dates. The distributions are very similar (extremely low K-S test statistic).

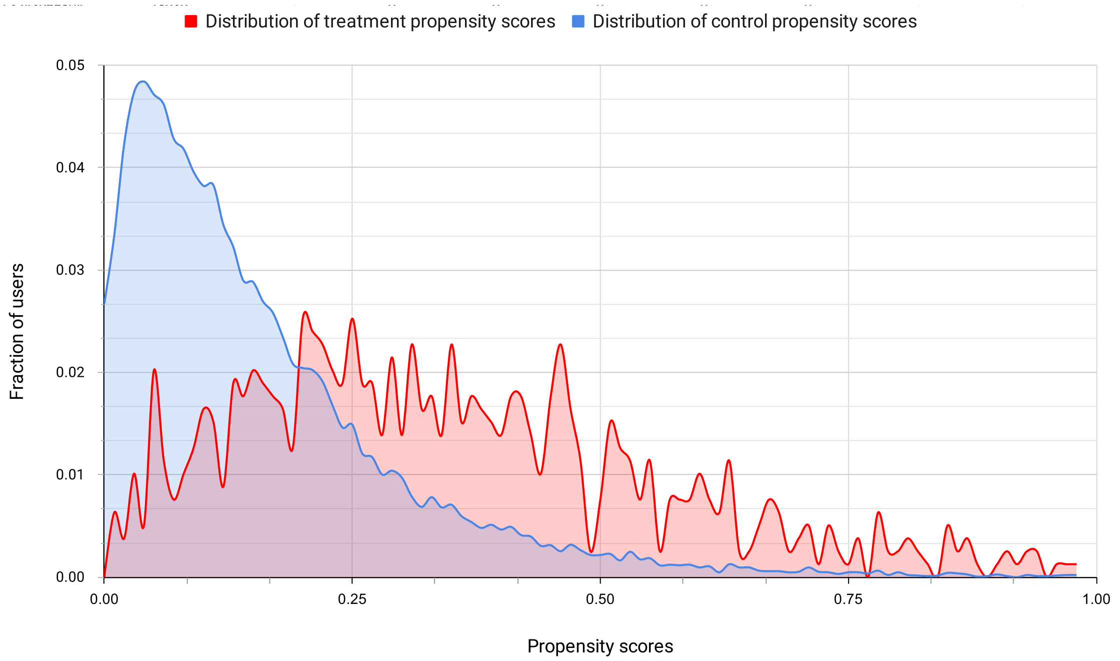

Figure 5: Distribution of propensity scores in the treatment and control groups. A considerable overlap between the propensity scores indicates that a causal study is viable, since users with similar covariates ended up in either of the groups.

Tables S1 to S3

Table 1: Descriptive statistics of our dataset.

Parameter	Statistic Type	Statistic Value
number of users under consideration	total	$43,832$
posts under consideration	from 2019	$19,132,467$
	from 2020 (till July 15)	$21,433,182$
	total	$40,565,649$
number of posts per user	mean $\pm\ \sigma$	929 ± 1156
	mode	59
followers per user	mean $\pm\ \sigma$	945 ± 2334
	median	286
	mode	16
	frequency of mode	185
followees per user	mean $\pm\ \sigma$	723 ± 829
	median	419
	mode	42
	frequency of mode	87

Table 2: Different categories of COVID-19 misinformation that were considered in this study.

	Misinformation Category
1.	"certain vitamin and food supplements helps in preventing or treating COVID-19"
2.	"gargling with warm water helps keep the coronavirus from traveling to the lungs"
3.	"the radiations emitted by the 5G technology causes COVID-19"
4.	"COVID-19 spreads from drinking water (including tap water in homes)"
5.	"the coronavirus was created as a biological weapon in Wuhan, China"
6.	"Bill Gates is responsible for the creation of the coronavirus"

Table 3: Randomly chosen examples of Twitter posts with different anxiety levels, as inferred by the anxiety scorer. The posts have been slightly rephrased to preserve anonymity of Twitter users; special symbols have been inserted to censor abusive words.

**Score Range**	**Twitter Posts**
0.75 – 1.00	→ "i hate having anxiety because of covid, it's literally killing me, i'm so done. sorry for complaining" → "i am in a constant state of despair and it is gonna kill me more than covid. i think of covid 24/7." → "covid anxiety is keeping me from enjoying life. how uncomfortable i am with my covid and the stupid f**k"
0.50 – 0.75	→ "my nervous system isn't courteous about when it fires up. it gets triggered at the grocery store, at the gym, in traffic. completely unpredictable lol. → "i truly do have a lot of nerve being as stressed out as i am and yet doing nothing to get anything done to relive said stress" → "am i wrong for feeling slightly uncomfortable and bummed about this situation cus idk i give my honest feelings and apparently maybe i'm wrong or the only one that feels this way"
0.25 – 0.50	→ "i get annoyed when people ask me for donations and funds for covid, black lives matter, folk artists, marginalized artists and climate change. oh the irony of it all!" →"idc what's opening during phase 1, i will let other people be the guinea pigs." → "just bc something helps you 'cope' doesn't automatically make it a good f**king thing to do"
0.00 – 0.25	→ "look at this brow lamination i did this week." → "i am a fan of his vocals. the enunciation, his intonation and how he carries off lower register. he's just so amazing" → "the way you breathe is the way you think. the way you think is the way you breathe."

Table 4: Distribution of sex and race in a sample of treatment and control groups.

		**Control Group (N = 1189)**	**Treatment Group (N = 762)**
**Sex**	Men	54.16%	60.10%
	Women	36.39%	30.71%
	Undetermined	9.45%	9.19%
**Race**	whites	37.91%	47.11%
	Blacks	12.85%	9.84%
	APIs	13.16%	8.92%
	Hispanics	10.10%	6.17%

References

[1] Twitter API documentation. `https://developer.twitter.com/en/docs/twitter-api`, 2021. Accessed: 06-25-2021.

[2] Jeremy Howard and Sebastian Ruder. Universal language model fine-tuning for text classification. In *Proceedings of the 56th Annual Meeting of the Association for Computational Linguistics (Volume 1: Long Papers)*, pages 328–339, 2018.

[3] Zhilin Yang, Zihang Dai, Ruslan Salakhutdinov, and William W Cohen. Breaking the softmax bottleneck: A high-rank rnn language model. *arXiv preprint arXiv:1711.03953*, 2017.

[4] Nicholas Micallef, Bing He, Srijan Kumar, Mustaque Ahamad, and Nasir Memon. The role of the crowd in countering misinformation: A case study of the COVID-19 infodemic. In *2020 IEEE International Conference on Big Data (Big Data)*, 2020.

[5] Munmun De Choudhury, Scott Counts, and Eric Horvitz. Social media as a measurement tool of depression in populations. In *Proceedings of the 5th Annual ACM Web Science Conference*, pages 47–56, 2013.

[6] Munmun De Choudhury, Emre Kiciman, Mark Dredze, Glen Coppersmith, and Mrinal Kumar. Discovering shifts to suicidal ideation from mental health content in social media. In *Proceedings of the 2016 CHI Conference on Human Factors in Computing Systems*, pages 2098–2110, 2016.

[7] Sharath Chandra Guntuku, Daniel Preotiuc-Pietro, Johannes C Eichstaedt, and Lyle H Ungar. What Twitter profile and posted images reveal about depression and anxiety. In *Proceedings of the International AAAI Conference on Web and Social Media*, volume 13, pages 236–246, 2019.

[8] Koustuv Saha, Benjamin Sugar, John Torous, Bruno Abrahao, Emre Kıcıman, and Munmun De Choudhury. A social media study on the effects of psychiatric medication use. In *Proceedings of the International AAAI Conference on Web and Social Media*, volume 13, pages 440–451, 2019.

[9] Koustuv Saha, John Torous, Eric D Caine, and Munmun De Choudhury. Psychosocial effects of the COVID-19 pandemic: Large-scale quasi-experimental study on social media. *Journal of Medical Internet Research*, 22(11):e22600, 2020.

[10] Guido W Imbens and Donald B Rubin. *Causal inference in statistics, social, and biomedical sciences.* Cambridge University Press, 2015.

[11] Donald B Rubin. Causal inference using potential outcomes: Design, modeling, decisions. *Journal of the American Statistical Association*, 100(469):322–331, 2005.

[12] Paul R Rosenbaum and Donald B Rubin. The central role of the propensity score in observational studies for causal effects. *Biometrika*, 70(1):41–55, 1983.

[13] James W Pennebaker, Martha E Francis, and Roger J Booth. Linguistic inquiry and word count: Liwc 2001. *Mahway: Lawrence Erlbaum Associates*, 71(2001):2001, 2001.

[14] Yla R Tausczik and James W Pennebaker. The psychological meaning of words: Liwc and computerized text analysis methods. *Journal of Language and Social Psychology*, 29(1):24–54, 2010.

[15] Emre Kiciman, Scott Counts, and Melissa Gasser. Using longitudinal social media analysis to understand the effects of early college alcohol use. In *Proceedings of the International AAAI Conference on Web and Social Media*, volume 12, 2018.

[16] Koustuv Saha, Asra Yousuf, Louis Hickman, Pranshu Gupta, Louis Tay, and Munmun De Choudhury. A social media study on demographic differences in perceived job satisfaction. *Proceedings of the ACM on Human-Computer Interaction (CSCW)*, 2021.

[17] Betty Pfefferbaum and Carol S North. Mental health and the COVID-19 pandemic. *New England Journal of Medicine*, 383(6):510–512, 2020.

[18] Matthew Wiswall and Basit Zafar. Preference for the workplace, investment in human capital, and gender. *The Quarterly Journal of Economics*, 133(1):457–507, 2018.

[19] Alan Mislove, Sune Lehmann, Yong-Yeol Ahn, Jukka-Pekka Onnela, and James Rosenquist. Understanding the demographics of Twitter users. In *Proceedings of the International AAAI Conference on Web and Social Media*, volume 5, 2011.

[20] Anikó Hannák, Claudia Wagner, David Garcia, Alan Mislove, Markus Strohmaier, and Christo Wilson. Bias in online freelance marketplaces: Evidence from TaskRabbit and Fiverr. In *Proceedings of the 2017 ACM Conference on Computer Supported Cooperative Work and Social Computing*, pages 1914–1933, 2017.

[21] Denae Ford, Alisse Harkins, and Chris Parnin. Someone like me: How does peer parity influence participation of women on Stack Overflow? In *2017 IEEE Symposium on Visual Languages and Human-Centric Computing (VL/HCC)*, pages 239–243. IEEE, 2017.

[22] Munmun De Choudhury, Sanket S Sharma, Tomaz Logar, Wouter Eekhout, and René Clausen Nielsen. Gender and cross-cultural differences in social media disclosures of mental illness. In *Proceedings of the 2017 ACM Conference on Computer Supported Cooperative Work and Social Computing*, pages 353–369, 2017.

[23] Aron Culotta, Nirmal Kumar, and Jennifer Cutler. Predicting the demographics of Twitter users from website traffic data. In *Proceedings of the AAAI Conference on Artificial Intelligence*, volume 29, 2015.

[24] Lucie Flekova, Daniel Preoţiuc-Pietro, and Lyle Ungar. Exploring stylistic variation with age and income on Twitter. In *Proceedings of the 54th Annual Meeting of the Association for Computational Linguistics (Volume 2: Short Papers)*, pages 313–319, 2016.

[25] Ashwin Rajadesingan, Reza Zafarani, and Huan Liu. Sarcasm detection on Twitter: A behavioral modeling approach. In *Proceedings of the Eighth ACM International Conference on Web Search and Data Mining*, pages 97–106, 2015.

[26] Paolo Fornacciari, Monica Mordonini, Agostino Poggi, Laura Sani, and Michele Tomaiuolo. A holistic system for troll detection on Twitter. *Computers in Human Behavior*, 89:258–268, 2018.

[27] Luo Si and Jamie Callan. A statistical model for scientific readability. In *Proceedings of the Tenth International Conference on Information and Knowledge Management*, pages 574–576, 2001.

[28] Clayton Allen Davis, Onur Varol, Emilio Ferrara, Alessandro Flammini, and Filippo Menczer. Botornot: A system to evaluate social bots. In *Proceedings of the 25th International Conference Companion on World Wide Web*, WWW '16 Companion, page 273–274, Republic and Canton of Geneva, CHE, 2016. International World Wide Web Conferences Steering Committee.

[29] Chengcheng Shao, Giovanni Luca Ciampaglia, Onur Varol, Kai-Cheng Yang, Alessandro Flammini, and Filippo Menczer. The spread of low-credibility content by social bots. *Nature Communications*, 9(1):1–9, 2018.

[30] Caleb Ziems, Bing He, Sandeep Soni, and Srijan Kumar. Racism is a virus: Anti-asian hate and counterhate in social media during the COVID-19 crisis. *arXiv preprint arXiv:2005.12423*, 2020.

[31] Adrian Rauchfleisch and Jonas Kaiser. The false positive problem of automatic bot detection in social science research. *PloS One*, 15(10):e0241045, 2020.

[32] Nikan Chavoshi, Hossein Hamooni, and Abdullah Mueen. Debot: Twitter bot detection via warped correlation. In *2016 IEEE 16th International Conference on Data Mining (ICDM)*, pages 817–822. IEEE Computer Society, 2016.

[33] Nikan Chavoshi, Hossein Hamooni, and Abdullah Mueen. On-demand bot detection and archival system. In *Proceedings of the 26th International Conference on World Wide Web Companion*, pages 183–187, 2017.

[34] Donald B Rubin. Randomization analysis of experimental data: The fisher randomization test comment. *Journal of the American Statistical Association*, 75(371):591–593, 1980.

[35] Michela Del Vicario, Alessandro Bessi, Fabiana Zollo, Fabio Petroni, Antonio Scala, Guido Caldarelli, H Eugene Stanley, and Walter Quattrociocchi. The spreading of misinformation online. *Proceedings of the National Academy of Sciences*, 113(3):554–559, 2016.

[36] Petter Törnberg. Echo chambers and viral misinformation: Modeling fake news as complex contagion. *PloS One*, 13(9):e0203958, 2018.

[37] Edward L Hannan. Randomized clinical trials and observational studies: guidelines for assessing respective strengths and limitations. *JACC: Cardiovascular Interventions*, 1(3):211–217, 2008.

[38] Sindhu Kiranmai Ernala, Michael L Birnbaum, Kristin A Candan, Asra F Rizvi, William A Sterling, John M Kane, and Munmun De Choudhury. Methodological gaps in predicting mental health states from social media: triangulating diagnostic signals. In *Proceedings of the 2019 CHI Conference on Human Factors in Computing Systems*, pages 1–16, 2019.
